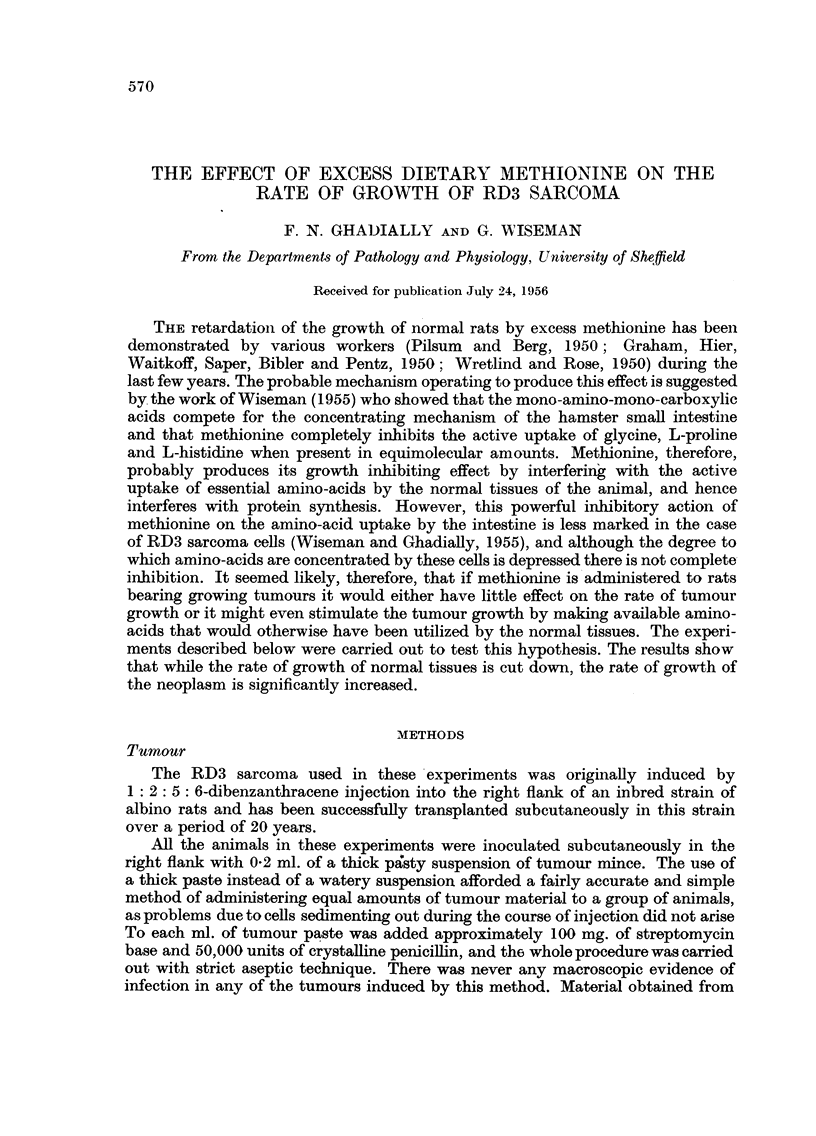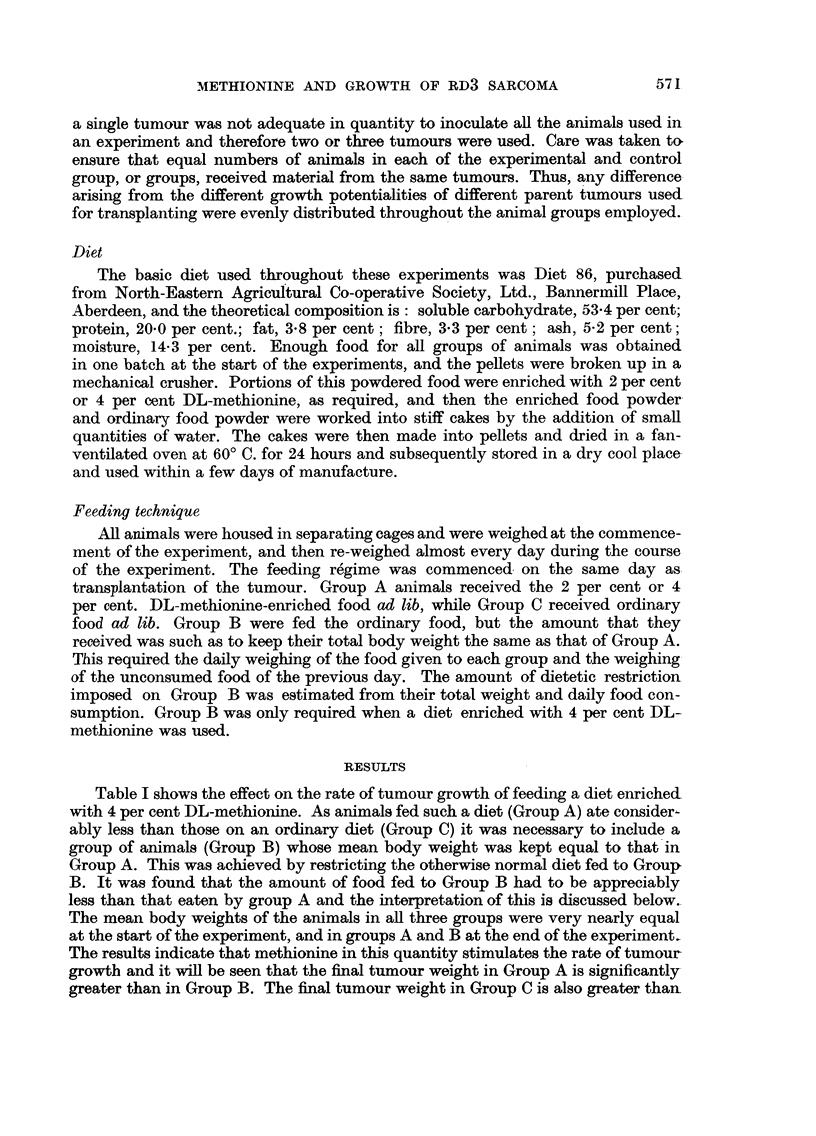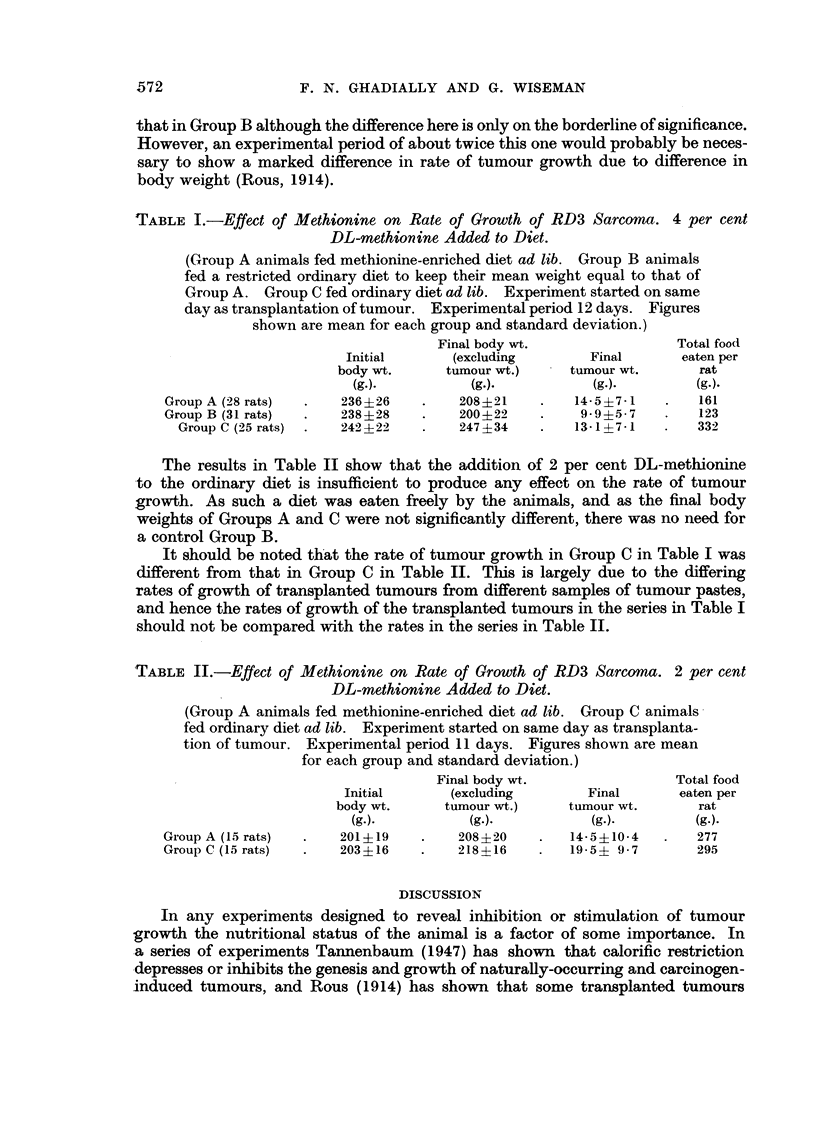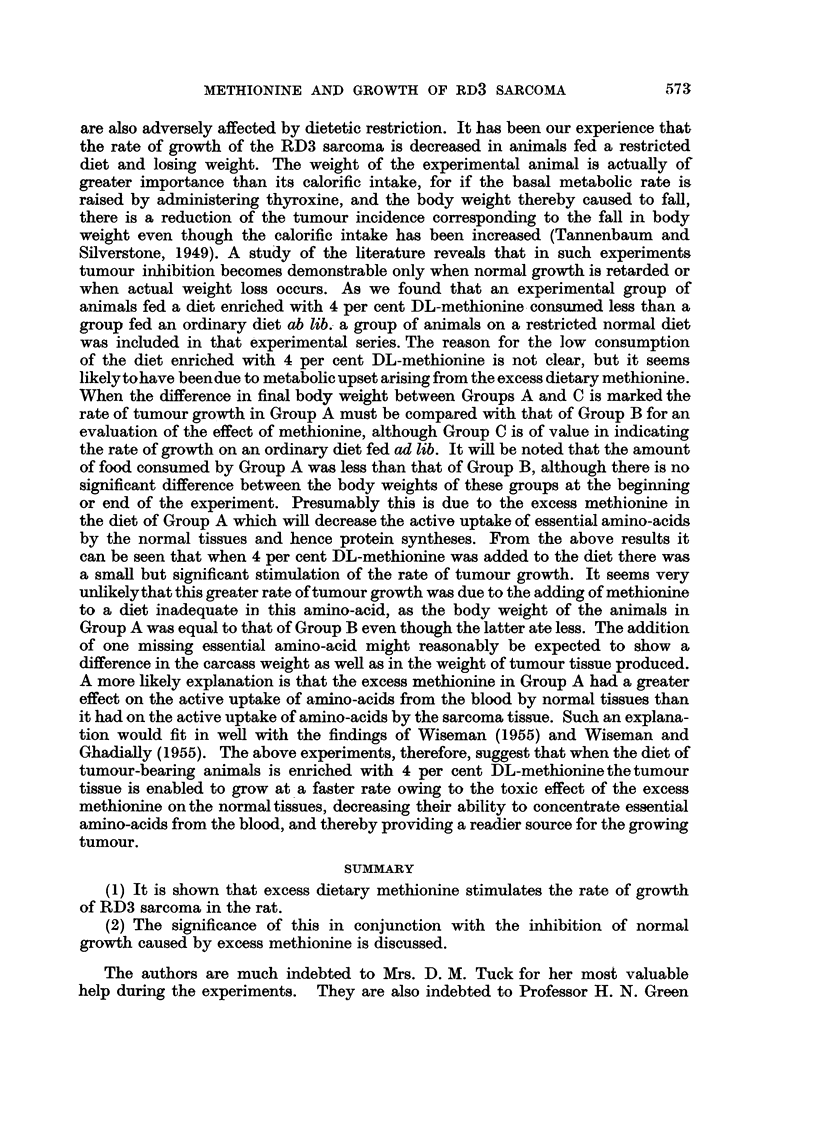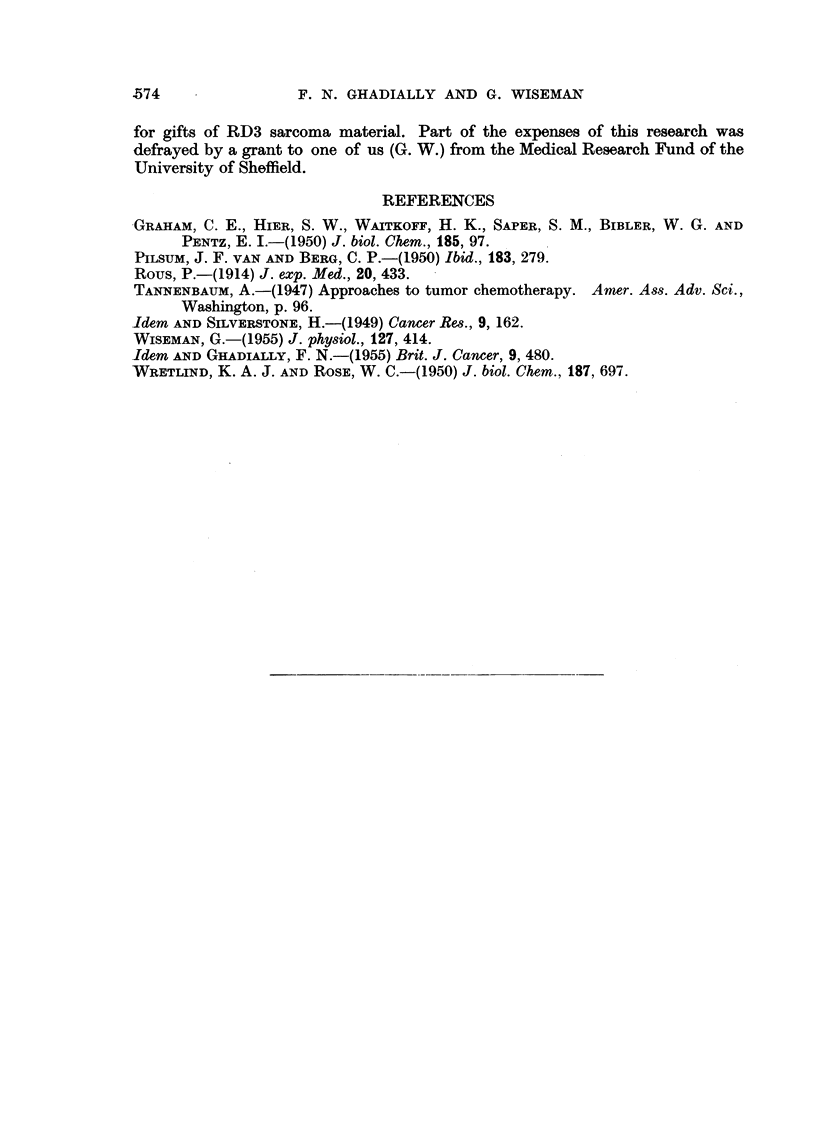# The Effect of Excess Dietary Methionine on the Rate of Growth of RD3 Sarcoma

**DOI:** 10.1038/bjc.1956.68

**Published:** 1956-09

**Authors:** F. N. Ghadially, G. Wiseman


### THE EFFECT OF EXCESS DIETARY METHIONINE ON THE RATE OF GROWTH OF RD3 SARCOMA

F. N. GHADIALLY AND G. WISEMAN

*From the Departments of Pathology and Physiology, University of Sheffield*

Received for publication July 24, 1956

THE retardation of the growth of normal rats by excess methionine has been demonstrated by various workers (Pilsum and Berg, 1950; Graham, Hier, Waitkoff, Saper, Bibler and Pentz, 1950; Wretlind and Rose, 1950) during the last few years. The probable mechanism operating to produce this effect is suggested by the work of Wiseman (1955) who showed that the mono-amino-mono-carboxylic acids compete for the concentrating mechanism of the hamster small intestine and that methionine completely inhibits the active uptake of glycine, L-proline and L-histidine when present in equimolecular amounts. Methionine, therefore, probably produces its growth inhibiting effect by interfering with the active uptake of essential amino-acids by the normal tissues of the animal, and hence interferes with protein synthesis. However, this powerful inhibitory action of methionine on the amino-acid uptake by the intestine is less marked in the case of RD3 sarcoma cells (Wiseman and Ghadially, 1955), and although the degree to which amino-acids are concentrated by these cells is depressed there is not complete inhibition. It seemed likely, therefore, that if methionine is administered to rats bearing growing tumours it would either have little effect on the rate of tumour growth or it might even stimulate the tumour growth by making available amino-acids that would otherwise have been utilized by the normal tissues. The experiments described below were carried out to test this hypothesis. The results show that while the rate of growth of normal tissues is cut down, the rate of growth of the neoplasm is significantly increased.

### METHODS

### *Tumour*

The RD3 sarcoma used in these experiments was originally induced by 1 : 2 : 5 : 6-dibenzanthracene injection into the right flank of an inbred strain of albino rats and has been successfully transplanted subcutaneously in this strain over a period of 20 years.

All the animals in these experiments were inoculated subcutaneously in the right flank with 0·2 ml. of a thick pasty suspension of tumour mince. The use of a thick paste instead of a watery suspension afforded a fairly accurate and simple method of administering equal amounts of tumour material to a group of animals, as problems due to cells sedimenting out during the course of injection did not arise To each ml. of tumour paste was added approximately 100 mg. of streptomycin base and 50,000 units of crystalline penicillin, and the whole procedure was carried out with strict aseptic technique. There was never any macroscopic evidence of infection in any of the tumours induced by this method. Material obtained from a single tumour was not adequate in quantity to inoculate all the animals used in an experiment and therefore two or three tumours were used. Care was taken to ensure that equal numbers of animals in each of the experimental and control group, or groups, received material from the same tumours. Thus, any difference arising from the different growth potentialities of different parent tumours used for transplanting were evenly distributed throughout the animal groups employed.

*Diet*

The basic diet used throughout these experiments was Diet 86, purchased from North-Eastern Agricultural Co-operative Society, Ltd., Bannermill Place, Aberdeen, and the theoretical composition is : soluble carbohydrate, 53·4 per cent; protein, 20·0 per cent.; fat, 3·8 per cent ; fibre, 3·3 per cent ; ash, 5·2 per cent ; moisture, 14·3 per cent. Enough food for all groups of animals was obtained in one batch at the start of the experiments, and the pellets were broken up in a mechanical crusher. Portions of this powdered food were enriched with 2 per cent or 4 per cent DL-methionine, as required, and then the enriched food powder and ordinary food powder were worked into stiff cakes by the addition of small quantities of water. The cakes were then made into pellets and dried in a fan-ventilated oven at 60° C. for 24 hours and subsequently stored in a dry cool place and used within a few days of manufacture.

*Feeding technique*

All animals were housed in separating cages and were weighed at the commencement of the experiment, and then re-weighed almost every day during the course of the experiment. The feeding régime was commenced on the same day as transplantation of the tumour. Group A animals received the 2 per cent or 4 per cent. DL-methionine-enriched food *ad lib,* while Group C received ordinary food *ad lib.* Group B were fed the ordinary food, but the amount that they received was such as to keep their total body weight the same as that of Group A. This required the daily weighing of the food given to each group and the weighing of the unconsumed food of the previous day. The amount of dietetic restriction imposed on Group B was estimated from their total weight and daily food consumption. Group B was only required when a diet enriched with 4 per cent DL-methionine was used.

### RESULTS

Table I shows the effect on the rate of tumour growth of feeding a diet enriched with 4 per cent DL-methionine. As animals fed such a diet (Group A) ate considerably less than those on an ordinary diet (Group C) it was necessary to include a group of animals (Group B) whose mean body weight was kept equal to that in Group A. This was achieved by restricting the otherwise normal diet fed to Group B. It was found that the amount of food fed to Group B had to be appreciably less than that eaten by group A and the interpretation of this is discussed below. The mean body weights of the animals in all three groups were very nearly equal at the start of the experiment, and in groups A and B at the end of the experiment. The results indicate that methionine in this quantity stimulates the rate of tumour growth and it will be seen that the final tumour weight in Group A is significantly greater than in Group B. The final tumour weight in Group C is also greater than that in Group B although the difference here is only on the borderline of significance. However, an experimental period of about twice this one would probably be necessary to show a marked difference in rate of tumour growth due to difference in body weight (Rous, 1914).

TABLE I.—*Effect of Methionine on Rate of Growth of RD3 Sarcoma. 4 per cent DL-methionine Added to Diet.*

(Group A animals fed methionine-enriched diet *ad lib.* Group B animals fed a restricted ordinary diet to keep their mean weight equal to that of Group A. Group C fed ordinary diet *ad lib.* Experiment started on same day as transplantation of tumour. Experimental period 12 days. Figures shown are mean for each group and standard deviation.)

| | Initial body wt. (g.). | Final body wt. (excluding tumour wt.) (g.). | Final tumour wt. (g.). | Total food eaten per rat (g.). |
|---|---|---|---|---|
| Group A (28 rats) | 236±26 | 208±21 | 14·5±7·1 | 161 |
| Group B (31 rats) | 238±28 | 200±22 | 9·9±5·7 | 123 |
| Group C (25 rats) | 242±22 | 247±34 | 13·1±7·1 | 332 |

The results in Table II show that the addition of 2 per cent DL-methionine to the ordinary diet is insufficient to produce any effect on the rate of tumour growth. As such a diet was eaten freely by the animals, and as the final body weights of Groups A and C were not significantly different, there was no need for a control Group B.

It should be noted that the rate of tumour growth in Group C in Table I was different from that in Group C in Table II. This is largely due to the differing rates of growth of transplanted tumours from different samples of tumour pastes, and hence the rates of growth of the transplanted tumours in the series in Table I should not be compared with the rates in the series in Table II.

TABLE II.—*Effect of Methionine on Rate of Growth of RD3 Sarcoma. 2 per cent DL-methionine Added to Diet.*

(Group A animals fed methionine-enriched diet *ad lib.* Group C animals fed ordinary diet *ad lib.* Experiment started on same day as transplantation of tumour. Experimental period 11 days. Figures shown are mean for each group and standard deviation.)

| | Initial body wt. (g.). | Final body wt. (excluding tumour wt.) (g.). | Final tumour wt. (g.). | Total food eaten per rat (g.). |
|---|---|---|---|---|
| Group A (15 rats) | 201±19 | 208±20 | 14·5±10·4 | 277 |
| Group C (15 rats) | 203±16 | 218±16 | 19·5± 9·7 | 295 |

### DISCUSSION

In any experiments designed to reveal inhibition or stimulation of tumour growth the nutritional status of the animal is a factor of some importance. In a series of experiments Tannenbaum (1947) has shown that calorific restriction depresses or inhibits the genesis and growth of naturally-occurring and carcinogen-induced tumours, and Rous (1914) has shown that some transplanted tumours are also adversely affected by dietetic restriction. It has been our experience that the rate of growth of the RD3 sarcoma is decreased in animals fed a restricted diet and losing weight. The weight of the experimental animal is actually of greater importance than its calorific intake, for if the basal metabolic rate is raised by administering thyroxine, and the body weight thereby caused to fall, there is a reduction of the tumour incidence corresponding to the fall in body weight even though the calorific intake has been increased (Tannenbaum and Silverstone, 1949). A study of the literature reveals that in such experiments tumour inhibition becomes demonstrable only when normal growth is retarded or when actual weight loss occurs. As we found that an experimental group of animals fed a diet enriched with 4 per cent DL-methionine consumed less than a group fed an ordinary diet *ab lib.* a group of animals on a restricted normal diet was included in that experimental series. The reason for the low consumption of the diet enriched with 4 per cent DL-methionine is not clear, but it seems likely to have been due to metabolic upset arising from the excess dietary methionine. When the difference in final body weight between Groups A and C is marked the rate of tumour growth in Group A must be compared with that of Group B for an evaluation of the effect of methionine, although Group C is of value in indicating the rate of growth on an ordinary diet fed *ad lib.* It will be noted that the amount of food consumed by Group A was less than that of Group B, although there is no significant difference between the body weights of these groups at the beginning or end of the experiment. Presumably this is due to the excess methionine in the diet of Group A which will decrease the active uptake of essential amino-acids by the normal tissues and hence protein syntheses. From the above results it can be seen that when 4 per cent DL-methionine was added to the diet there was a small but significant stimulation of the rate of tumour growth. It seems very unlikely that this greater rate of tumour growth was due to the adding of methionine to a diet inadequate in this amino-acid, as the body weight of the animals in Group A was equal to that of Group B even though the latter ate less. The addition of one missing essential amino-acid might reasonably be expected to show a difference in the carcass weight as well as in the weight of tumour tissue produced. A more likely explanation is that the excess methionine in Group A had a greater effect on the active uptake of amino-acids from the blood by normal tissues than it had on the active uptake of amino-acids by the sarcoma tissue. Such an explanation would fit in well with the findings of Wiseman (1955) and Wiseman and Ghadially (1955). The above experiments, therefore, suggest that when the diet of tumour-bearing animals is enriched with 4 per cent DL-methionine the tumour tissue is enabled to grow at a faster rate owing to the toxic effect of the excess methionine on the normal tissues, decreasing their ability to concentrate essential amino-acids from the blood, and thereby providing a readier source for the growing tumour.

### SUMMARY

(1) It is shown that excess dietary methionine stimulates the rate of growth of RD3 sarcoma in the rat.

(2) The significance of this in conjunction with the inhibition of normal growth caused by excess methionine is discussed.

The authors are much indebted to Mrs. D. M. Tuck for her most valuable help during the experiments. They are also indebted to Professor H. N. Green for gifts of RD3 sarcoma material. Part of the expenses of this research was defrayed by a grant to one of us (G. W.) from the Medical Research Fund of the University of Sheffield.

### REFERENCES

GRAHAM, C. E., HIER, S. W., WAITKOFF, H. K., SAPER, S. M., BIBLER, W. G. AND PENTZ, E. I.—(1950) *J. biol. Chem.*, **185**, 97.
PILSUM, J. F. VAN AND BERG, C. P.—(1950) *Ibid.*, **183**, 279.
ROUS, P.—(1914) *J. exp. Med.*, **20**, 433.
TANNENBAUM, A.—(1947) Approaches to tumor chemotherapy. *Amer. Ass. Adv. Sci.*, Washington, p. 96.
*Idem* AND SILVERSTONE, H.—(1949) *Cancer Res.*, **9**, 162.
WISEMAN, G.—(1955) *J. physiol.*, **127**, 414.
*Idem* AND GHADIALLY, F. N.—(1955) *Brit. J. Cancer*, **9**, 480.
WRETLIND, K. A. J. AND ROSE, W. C.—(1950) *J. biol. Chem.*, **187**, 697.